# Therapeutic Potential of Photosynthetic Microorganisms for Visceral Leishmaniasis: An Immunological Analysis

**DOI:** 10.3389/fimmu.2022.891495

**Published:** 2022-06-30

**Authors:** Victor Vaitkevicius-Antão, Jady Moreira-Silva, Isabelle Barreto da Silva Moreira Reino, Maria Gabriella Nunes de Melo, José Noé da Silva-Júnior, Alexsandra Frazão de Andrade, Paulo Sérgio Ramos de Araújo, Raquel Pedrosa Bezerra, Daniela de Araújo Viana Marques, Silvana Ferreira, Rômulo Pessoa-e-Silva, Virginia Maria Barros de Lorena, Milena de Paiva-Cavalcanti

**Affiliations:** ^1^ Department of Microbiology, Aggeu Magalhães Institute, Fiocruz Pernambuco, Recife, Brazil; ^2^ Research Support Center, Federal Rural University of Pernambuco, Recife, Brazil; ^3^ Institute of Biological Sciences, University of Pernambuco, Recife, Brazil; ^4^ Central Public Health Laboratory of Pernambuco, Endemics Laboratory, Recife, Brazil; ^5^ Department of Immunology, Aggeu Magalhães Institute, Fiocruz Pernambuco, Recife, Brazil

**Keywords:** cyanobacteria, *Leishmania infantum*, microalgae, immunomodulation, therapy

## Abstract

New therapeutic strategies for visceral leishmaniasis (VL) have been studied, and the development of an immunotherapeutic agent that modulates the host’s immune response is necessary. The aim of this study was to evaluate *in vitro* the bioactive extracts of photosynthetic microorganisms (PMs) for their leishmanicidal/leishmanistatic and immunomodulatory potentials. Bioactive extracts from PMs (*Arthrospira platensis* and *Dunaliella tertiolecta*) were obtained by sonication. Reference drugs, miltefosine (MTF) and N-methylglucamine antimoniate (Sb^V^), were also evaluated. The selectivity index (SI) of treatments was determined by assays of inhibitory concentration (IC_50_) in *Leishmania infantum* cells and cytotoxic concentrations (CC_50_) in human peripheral blood mononuclear cells by the MTT method. The immune response was evaluated in healthy human cells by the production of cytokines and nitric oxide (NO) and the gene expression of *Tbx21*, *GATA3*, *RORc*, and *FOXP3*, using four concentrations (CC_50_, ½ CC_50_, ¼ CC_50_, and IC_50_) for *in-vitro* stimulation. Based on the data obtained, we observed that the extracts of *D. tertiolecta* (SI = 4.7) and *A. platensis* (SI = 3.8) presented better results when compared to Sb^V^ (SI = 2.1). When analyzing the immune response results, we identified that the extracts of PMs stimulated the production of cytokines of the Th1 profile more than the reference drugs. The extracts also demonstrated the ability to stimulate NO synthesis. Regarding gene expression, in all concentrations of *A. platensis* extracts, we found a balance between the Th1/Th2 profile, with the average expression of the *Tbx21* gene more than the *GATA3* in the highest concentration (CC_50_). Regarding the extract of *D. tertiolecta*, we can observe that, in the lowest concentrations, a balance between all the genes was present, with the average expression of the *GATA3* gene being lower than the others. The best result was found in the ½ CC_50_ concentration, stimulating a balanced positive expression between the Th1×Th17×Treg profiles, with a negative expression of *GATA3*. Thus, PM extracts showed promising results, presenting low toxicity, leishmanicidal/leishmanistatic activity, and induction of the immune response, which could be potential therapeutic candidates for VL.

## Introduction

The lack of therapeutic alternatives is one of the greatest obstacles to control visceral leishmaniasis (VL). In the treatment of VL, the drug of first choice for humans is N-methylglucamine antimoniate (Glucantime^®^, Sanofi-Aventis, France), and for dogs, it is miltefosine (Milteforan™, Virbac, France). However, these drugs are highly toxic to the host and have been presenting treatment failures and reports of parasite resistance (1).

The host’s immune response against the parasite must change, at least in part, to achieve therapeutic success against VL; in this context, the use of immunomodulatory drugs that can reverse the host’s immune profile, stimulating a profile of resistance against the parasite, is important to complement conventional treatment, helping in the patient’s clinical improvement (2). To investigate the protective immune response stimulated by a therapeutic candidate, understanding the immunological mechanisms in leishmaniasis is essential, as these parasites have developed, throughout evolution, several evasion mechanisms to survive the host’s immune system (3).

The host’s cellular immune response is mediated by CD4^+^ T lymphocytes which, in a simplified way, can present two types of profiles: resistance (Th1) and susceptibility (Th2). The Th1 profile produces cytokines such as interferon-gamma (IFN-γ), tumor necrosis factor (TNF), and interleukin 2 (IL-2), which are associated with the control of infection by macrophages, the production of nitric oxide (NO), and the consequent destruction of intracellular parasites. On the other hand, the Th2 profile is characterized by the synthesis of cytokines such as interleukin 4 (IL-4), interleukin 10 (IL-10), and tumor growth factor-beta (TGF-β), favoring the onset of the disease and its symptoms, with inhibition of synthesized NO by activated macrophages, providing the survival of the parasites (4–7).

Other cell subtypes such as regulatory T cells (Treg) and Th17 may also play a significant role in susceptibility and resistance against leishmaniasis (8, 9). However, despite the Th1/Th2 (resistance/susceptibility) paradigm being a simplified model, several studies have demonstrated its foundation (10–12).

Based on this knowledge about the immunological profile of VL, studies that aim to investigate new molecules with potential against visceral leishmaniasis can be performed. Microorganisms, such as photosynthetic microorganisms, are promising candidates for therapeutic investigations since they have a variety of metabolites with different biological activities that have not been fully studied yet (13–15). Studies with Chlorophyta extracts have demonstrated anti-inflammatory and antiparasitic activities (16, 17), in addition to an immunomodulatory potential (18). Thus, with a broader vision for the control of leishmaniasis, this study aimed to evaluate the *in-vitro* therapeutic potential for VL of the bioactive extracts of two species of photosynthetic microorganisms, *Arthrospira platensis* and *Dunaliella tertiolecta*, in human cells.

## Material and Methods

### Obtaining Bioactive Extracts of Photosynthetic Microorganisms

Two photosynthetic microorganisms (PMs), the cyanobacterium *A. platensis* (Utex, 1926) and the microalgae *D. tertiolecta* (Utex, 1644), were obtained from the University of Texas culture collection. *Arthrospira platensis* was cultivated in a 250-ml Erlenmeyer flask containing 100 ml of Schlösser medium (19) in an orbital shaker at 75 ± 5 rpm, while *D. tertiolecta* was cultivated in a 1-L Erlenmeyer flask containing 400 ml of f/2 medium (20), under constant aeration. Both cultivations used an initial biomass concentration of 50 mg/L, temperature of 27°C ± 1°C, and continuous light intensity of 52 ± 4 μmol photons m^−2^ s^−1^ (21).

After reaching the stationary growth phase, the cultures were centrifuged at 430×*g* for 10 min (Hermle Labortechnik 326 HK, Wehingen, Germany), lyophilized (SP Scientific BenchTop Pro, Warminster, USA), resuspended (100 mg ml^−1^) in 0.1 M of Tris–HCl buffer pH 7.0, and sonicated at 20 pulses in an ice bath with intervals of 1 min between each pulse (22). The homogenate was centrifuged at 10,000×*g* for 5 min at 4°C, and the supernatant filtered at 0.22 µm was denominated bioactive extract and used for further analysis. Protein concentration was determined by the method of Smith et al. (23) using the Micro BCA Protein Assay Kit (Pierce).

### Cultivation of *Leishmania infantum*

The promastigotes of *L. infantum* (strain MHOM/BR/1972/BH46) were culture-expanded in Schneider medium (Sigma-Aldrich) pH 7.2, containing 10% of fetal bovine serum (FBS—Vitrocell^®^, Brazil) and 1% of antibiotic (penicillin 200 U/ml). The peaks were performed between the third and fourth day of cultivation and incubated in a biochemical oxygen demand greenhouse (BOD) at a temperature of ±26°C, to be used in the leishmanicidal effect tests.

To obtain the antigenic fractions, the parasitic mass was subjected to rapid freezing (−197°C) in liquid nitrogen, followed by thawing (40°C). Soon after, the cell suspension was subjected to the ultrasonication process (60 s/40 W), three times, and centrifuged at 10,000×*g* for 20 min at 4°C. The resulting supernatant was a soluble antigen fraction (*L. infantum* soluble antigen: LSA). Proteins were quantified by the Bradford method (24), and the fraction was stored at −80°C. LSA was used in the cell culture assays.

### Sample Collection and Peripheral Blood Mononuclear Cell Isolation

Whole blood was collected from healthy human individuals by venipuncture using heparin tubes (18 ml) to perform the separation of peripheral blood mononuclear cells (PBMCs). Phosphate-buffered saline (PBS) (pH 7.2) was added to heparinized blood in a proportion of 1:2. This mixture was added to Ficoll-Hypaque at a ratio of 1:3 (Ficoll-Hypaque:blood) to obtain the ring of PBMCs after centrifugation at 900×*g* for 30 min without brake. Cells were washed twice in PBS and then resuspended in 1 ml of RPMI 1640 medium supplemented with 10% of FBS and 1% of penicillin/streptomycin (200 U/ml). Cells were counted in a Neubauer chamber using Trypan Blue dye. The cell value obtained was adjusted to a concentration of 1 × 10^6^ cells/ml by adding RPMI 1640 medium supplemented with 10% of FBS, to be used in the assays.

### Determination of the Selectivity Index

The selectivity index (SI) was calculated as the ratio between the 50% cytotoxic concentration (CC_50_) in human cells and the 50% inhibitory concentration (IC_50_) in parasite cultures. All cells were treated with bioactive extracts of photosynthetic microorganisms and reference drugs (N-methylglucamine antimoniate and miltefosine).

PBMCs from healthy humans were cultivated in 96-well plates and treated with different stimuli to evaluate the CC_50_. Cell treatment (containing 1 × 10^6^ cells/ml) was performed in triplicate for each stimulus, incubated for 24 h, using concentrations of 62.5 to 1,000 µg/ml of bioactive extracts of PMs (25, 26). After the culture time, the supernatant was discarded and MTT [3-(4,5-dimethylthiazol-2-yl)-2,5-diphenyltetrazolium bromide] was added at a concentration of 1 mg/ml, solubilized in RPMI 1640 medium without phenol red, and incubated in an oven at 37°C for 3 h. Subsequently, the formazan crystals formed were solubilized with dimethylsulfoxide (DMSO) and then read in a spectrophotometer at 540 nm (27).

Cells of *L. infantum* in the promastigote form were cultivated for the evaluation of the IC_50_ of the PM bioactive extracts. Cell treatment was performed in triplicate for each stimulus, incubated for 48 h in a 96-well plate containing 1 × 10^6^ cells/ml (28). After the culture time, the cells were counted and evaluated for their morphology under a light microscope. For natural extracts, protein concentrations of 15.63 to 500 µg/ml were used (29). For N-methylglucamine antimoniate and miltefosine, the concentration curves were 15.63 to 250 µg/ml and 0.1 to 4 µg/ml, respectively (30, 31). Three independent experiments were carried out in triplicates.

### Immune Response Analysis

Whole blood was collected from healthy humans to obtain the PBMCs. PBMC cultures were performed in 48-well polystyrene plates, containing 5 × 10^5^ cells/well, and stimulated with the mitogen phytohemagglutinin (PHA) (10 µg/ml) (positive control of the culture), the soluble antigen of *L. infantum* (LSA) (25 µg/ml) (32), N-methylglucamine antimoniate, miltefosine, and natural extracts. The concentration values—CC_50_, ½ CC_50_, ¼ CC_50_, and IC_50_—of the reference drugs and natural extracts were determined. Unstimulated wells served as a negative control.

#### Evaluation of Cytokine Production

From the supernatants of human PBMC cultures, cytokine secretion (IFN-γ, TNF, IL-2, IL-4, IL-6, IL-10) was measured using the BD™ CBA Human Th1/Th2 Cytokine kit (Becton Dickinson, United States). The reading was performed in the FACSCalibur flow cytometry equipment (Becton Dickinson, United States), according to the manufacturer’s guidelines. The results were analyzed using the FCAP Array 3.0 software (Becton Dickinson, United States), being normalized with the results obtained by non-stimulated cells. So, if the value is presented as negative, it means that there is an inhibition of cytokine production since the detected value is lower than that presented by the non-stimulated PBMCs.

#### Evaluation of Nitric Oxide Production

Quantification of NO levels was indirectly performed by measuring nitrite in culture supernatants, by the Griess reaction, according to Resende et al. (7). The stimuli (LSA, N-methylglucamine antimoniate, miltefosine, and natural extracts) were compared with the negative control (PBMCs without stimulus).

#### Evaluation of mRNA Expression

The evaluation of the relative expression of messenger RNA (mRNA) of specific transcription factors (Tbx21, GATA3, RORc, and FOXP3) from the culture of healthy human PBMCs was performed. After the cultivation time, RNA extraction was performed with the TRIzol^®^ (Invitrogen, Thermo Fisher Scientific, United States) method, measuring the mRNA concentration in a spectrophotometer and storing it at −80°C.

After obtaining the mRNA, reverse transcription to complementary DNA (cDNA) was performed using the commercial TaqMan Reverse Transcription reagent kit (Applied Biosystems, Life Technology, United States). The cycling conditions followed the manufacturer’s guidelines (25°C for 10 min, 37°C for 30 min, 95°C for 5 min, 4°C until removal).

A real-time qPCR was performed from the cDNA (TaqMan^®^ System, Applied Biosystems, Life Technology, United States) using the QuantStudio 5 sequence detection system and the TaqMan^®^ Gene Expression PCR Master Mix kit (Applied Biosystems, Life Technology, United States) for the main regulatory genes *T-bet*/*Tbx21* (ID: Hs00894392_m1), *GATA3* (ID: Hs00231122_m1), *RORc* (ID: Hs01076112_m1), and *FOXP3* (ID: Hs01085834_m1). As an endogenous control of the reaction, *GAPDH* was used (ID: Hs02786624_g1). All samples were analyzed in duplicate and expressed as the mean ± standard error of the mean, using the PHA mitogen as a calibrator to calculate the relative quantification value.

### Data Analysis

Probit regression analyses were performed using the IBM SPSS Statistic 25 software program in order to determine the values of CC_50_ and IC_50_. Also, comparative analyses were performed using descriptive statistics techniques with absolute and percentage distributions. The Kolmogorov–Smirnov normality test was used, and according to the analysis, Student’s *t*-test (parametric) or the Mann–Whitney test (non-parametric) was performed for comparisons between two categories, or one-way analysis of variance (ANOVA) with Tukey post-test (parametric) or Kruskal–Wallis with Dunn’s post-test (non-parametric) was performed for comparisons between more than two categories. Correlation analyses were also performed using Pearson’s test (parametric) and Spearman’s test (non-parametric). All tests were performed using the GraphPad Prism 5 program (GraphPad Software Inc. 2007, San Diego, CA, USA), and a *p*-value <0.05 was considered significant.

### Ethical Considerations

Before the collection, human individuals included in the study were invited to participate and sign an informed consent form, authorizing the use of the collected material for scientific purposes. This study is part of the project “Immunology applied to the development of new control strategies for leishmaniasis” which has the approval of the Research Ethics Committee: CAAE 89972718.8.0000.5190 No. 4,077,060 (Fiocruz/PE).

## Results

### Determination of the Selectivity Index

Assays for the determination of CC_50_ and IC_50_ were performed, and it was observed that the extract of *A. platensis* had the lowest toxicity for human PBMCs, presenting a CC_50_ of approximately 1,000 µg/ml. In turn, *D. tertiolecta* presented higher toxicity to *L. infantum*, with a lower IC_50_.

By analyzing the reference drugs Sb^V^ and MTF, we observed that MTF was more toxic to *L. infantum* cells as well as to the host cells. Despite the high toxicity, the MTF was over 100 times more selective than other treatments (SI = 129.6). On the other hand, Sb^V^ had the lowest SI (2.11), demonstrating that the extracts of *A. platensis* and *D. tertiolecta* were more selective for the parasite (SI = 3.8 and 4.7, respectively) than this reference drug, which is currently used for the treatment of VL in Brazil. **Table 1** shows the CC_50_ and IC_50_ values as well as the SI.

**Table 1:** Leishmanicidal activity (IC_50_) in promastigotes and cytotoxic effect (CC_50_) in human cells treated with bioactive extracts of photosynthetic microorganisms and reference drugs.

Treatment	IC_50_	CC_50_	SI
	*L. infantum* (µg/ml)	Human PBMCs (µg/ml)	CC_50_/IC_50_
*A. platensis*	259.20	986.11	3.80
*D. tertiolecta*	53.75	252.55	4.70
Sb^V^	195.15	412.46	2.11
Miltefosine	1.23	159.49	129.6

SI, selectivity index; PBMCs, peripheral blood mononuclear cells; Sb^V^, N-methylglucamine antimoniate.

### Cytokine Quantification

**Figure 1:**
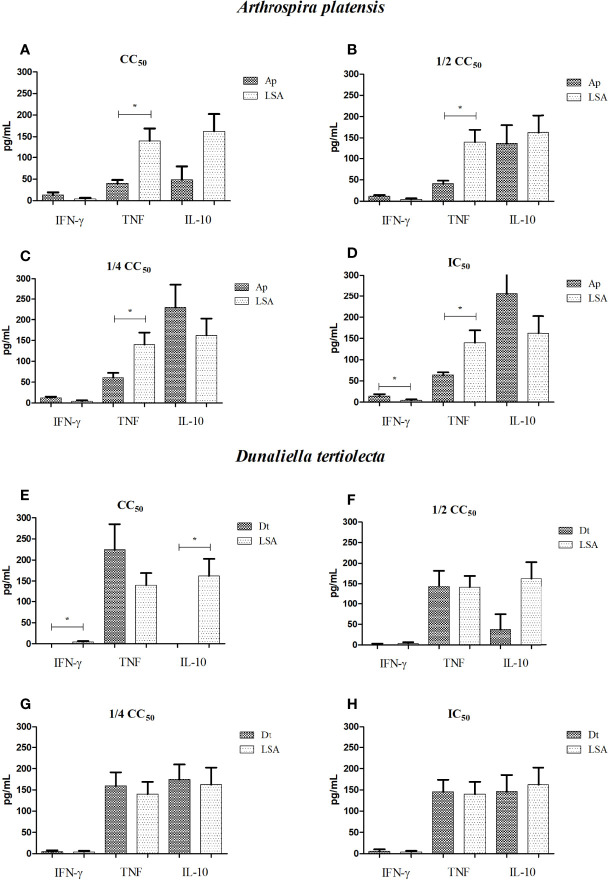
Dosage of cytokines produced by cells stimulated with bioactive extracts of photosynthetic microorganisms. **(A, E)** CC_50_ value; **(B, F)** half of the CC_50_ value; **(C, G)** one quarter of the CC_50_ value; **(D, H)** IC_50_ value. LSA, *Leishmania infantum* soluble antigen; Ap, *Arthrospira platensis*; Dt, *Dunaliella tertiolecta*. *Statistical difference = *p*-value < 0.05. Limit of detection (IFN-γ: 7.1; TNF: 2.8; IL-10: 2.8). The cytokine produced for each treatment was subtracted by the cytokine produced from non-stimulated cells.

For the extracts, we observed that both *A. platensis* and *D. tertiolecta* induced the production of cytokines of the Th1 profile by PBMCs, at all concentrations evaluated, demonstrating a positive stimulus against VL (**Figure 1**). We emphasized IL-10, which gradually decreased its production as the concentration of the extracts increased, with statistically significant differences between the concentrations of the *A. platensis* (*p*-value = 0.031) and *D. tertiolecta* (*p*-value = 0.007) extracts.

Comparing the results obtained from the extract of *A. platensis* with LSA, we observed the presence of a Th1-protective response for all concentrations, with IFN-γ stimulation higher than that observed with the natural infection mimicked by LSA, with a statistically significant difference for the IC_50_ concentration (*p*-value = 0.032) (**Figure 1D**). For all concentrations (**Figures 1A–D**), the *A. platensis* extract did not stimulate TNF production that was superior to LSA, with statistically significant differences, but it stimulated the production of this cytokine more than the cells without stimulation. Regarding the extract of *D. tertiolecta*, compared to LSA, we detected that, for all concentrations, a Th1-protective response was found, with the mean of TNF stimulation higher than that observed with LSA (**Figures 1E–H**). Furthermore, at the concentration of CC_50_, a lower stimulus for IL-10 production (*p*-value = 0.002) and for IFN-γ (*p*-value = 0.008) was found than LSA (**Figure 1E**). **Table 2** shows the mean values of cytokine production (in pg/ml) after cell stimulation with the four concentrations of each extract.

**Table 2:** Mean (± SEM) concentrations (in pg/ml) of cytokines produced by cells stimulated with the extracts of *Arthrospira platensis* and *Dunaliella tertiolecta*.

Cytokine	CC_50_	½ CC_50_	¼ CC_50_	IC_50_	*p*-value
*A. platensis*					
IFN-γ	13.63 ± 5.77	11.90 ± 2.45	12.54 ± 2.79	14.78 ± 4.18	0.92
TNF	40.71 ± 7.44	41.27 ± 6.77	61.19 ± 11.25	64.18 ± 6.18	0.63
IL-10	48.77 ± 30.91	137.2 ± 42.81	229.9 ± 55.75	255.7 ± 58.40	0.031*
IL-4	0.21 ± 0.09	0.33 ± 0.14	−0.03 ± 0.09	0.18 ± 0.13	0.21
IL-2	−1.03 ± 0.24	−0.46 ± 0.56	−1.20 ± 0.31	−0.72 ± 0.13	0.48
*D. tertiolecta*					
IFN-γ	−3.502 ± 2.93	0.674 ± 2.75	4.542 ± 3.01	5.504 ± 4.58	0.20
TNF	225.0 ± 59.88	143.3 ± 38.05	160.2 ± 31.57	146.0 ± 28.23	0.48
IL-10	−21.48 ± 29.12	37.85 ± 36.79	175.1 ± 34.85	146.3 ± 38.75	0.007*
IL-4	−0.038	0.20	−0.148	0.142	0.51
IL-2	−0.942	−0.43	−0.704	−1.074	0.43

Limit of detection (IFN-γ: 7.1 pg/ml; TNF: 2.8 pg/ml; IL-10: 2.8 pg/ml; IL-4: 2.6 pg/ml; IL-2: 2.6 pg/ml). Five independent experiments were performed with different individuals. The cytokine produced for each treatment was subtracted by the cytokine produced from non-stimulated cells.

CC_50_, 50% cytotoxic concentration; IC_50_, 50% inhibitory concentration.

*Statistical difference = p-value <0.05 compared among the concentrations.

When analyzing the correlation of cytokines and the extracts, we observed that between TNF and IL-4, in the CC_50_ and ½ CC_50_ concentrations of the *A. platensis* extract, there were a very strong and significant negative correlation (*r* = −0.92; *p*-value = 0.02) and a significant positive correlation (*r* = −0.97; *p*-value = 0.01), respectively. For the extract of *D. tertiolecta*, we observed very strong but not significant correlations. **Supplementary Table 1** shows the correlation data of cytokines induced by the extracts.

Regarding the reference drugs, all concentrations of Sb^V^, except IC_50_, showed an immunological suppression, with only IL-10 being stimulated. As for MTF, all concentrations, except IC_50_, showed immunological suppression, with stimulation of only TNF at the ¼ CC_50_ concentration. **Table 3** shows the mean values of cytokine production stimulated by the concentrations of each drug. Regarding the correlation between cytokines, for Sb^V^, there were very strong but not significant correlations. For MTF, we observed very strong and significant negative correlations between IL-4 and IL-2 in the CC_50_ concentration (*r* = 0.90; *p*-value = 0.03) and between TNF and IL-10 in the IC_50_ concentration (*r* = 0.91; *p*-value = 0.01). **Supplementary Table 2** shows the correlation data of cytokines induced by the reference drugs.

**Table 3:** Means of concentrations (in pg/ml) of cytokines produced by cells stimulated with the reference drugs.

Cytokine	CC_50_	½ CC_50_	¼ CC_50_	IC_50_
N-methylglucamine antimoniate				
IFN-γ	−2.853	−3.743	−1.335	2.000
TNF	−2.603	−0.300	−0.418	0.900
IL-10	8.112	2.213	4.268	11.82
IL-4	−0.398	−0.403	−0.288	0.298
IL-2	−1.138	−1.395	−0.720	−0.640
Miltefosine				
IFN-γ	−4.660	−4.635	−3.998	1.538
TNF	−8.105	−4.945	0.635	−2.138
IL-10	−35.08	−35.16	−34.54	0.128
IL-4	−0.263	−0.39	−0.185	−0.273
IL-2	−1.670	−1.748	−1.508	−1.138

Limit of detection (IFN-γ: 7.1 pg/ml; TNF: 2.8 pg/ml; IL-10: 2.8 pg/ml; IL-4: 2.6 pg/ml; IL-2: 2.6 pg/ml). Five independent experiments were performed with different individuals. The cytokine produced for each treatment was subtracted by the cytokine produced from non-stimulated cells.

CC_50_, 50% cytotoxic concentration; IC_50_, 50% inhibitory concentration.

### Quantification of Nitric Oxide

**Figure 2:**
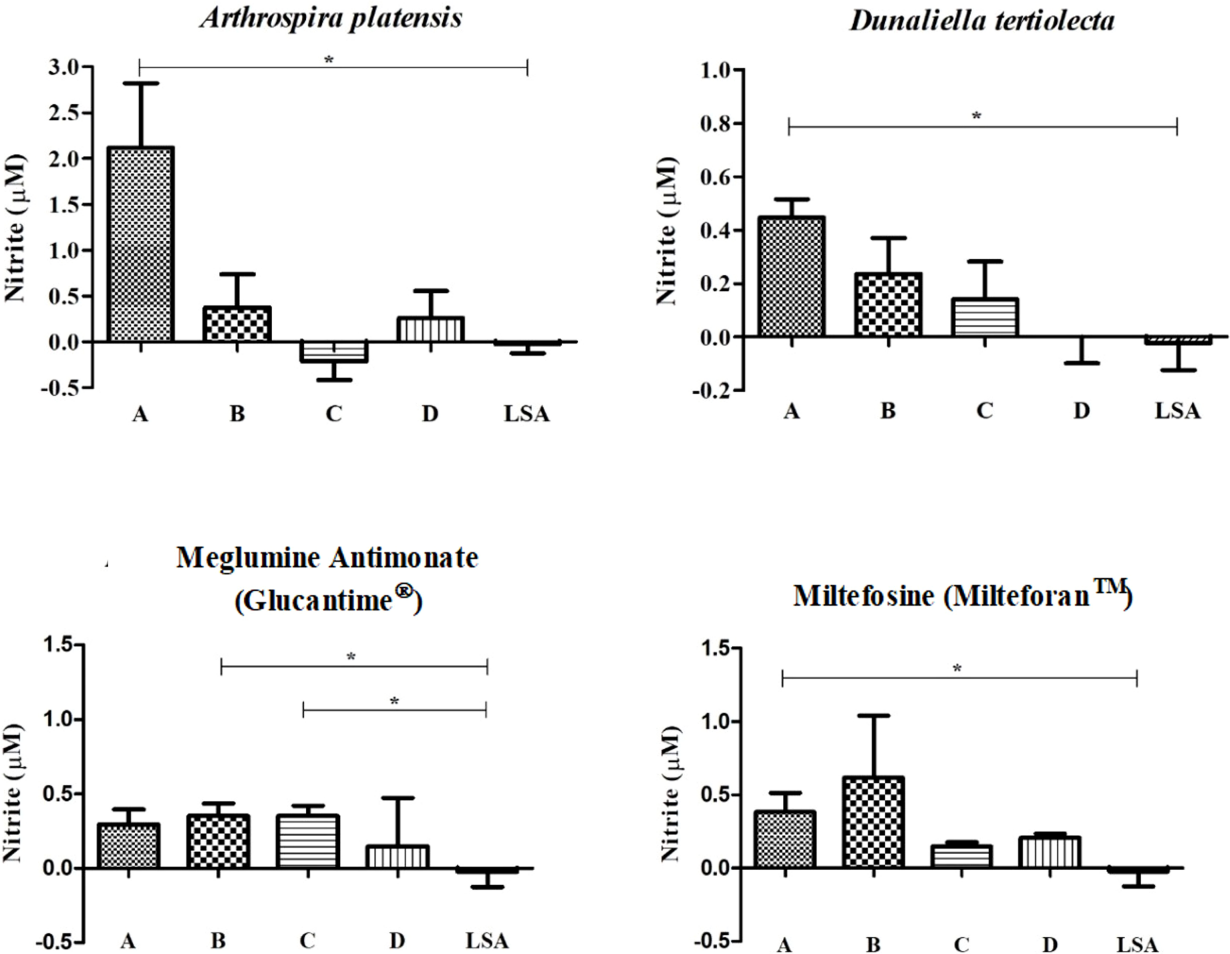
Measurement of nitrite after cell stimulation with bioactive extracts of photosynthetic microorganisms. LSA, soluble antigen of Leishmania infantum (25 µg/ml). *Statistical difference = p-value < 0.05. Limit of detection (0.055). The nitrite measured for each treatment was subtracted by the nitrite measured from non-stimulated cells.

For the extracts, we observed a slightly higher NO production when compared to cells without stimulus and stimulated with LSA (which demonstrated a reduction in NO production, when compared to cells without stimulus). We also identified that the nitrite dosage was directly proportional to the stimulus concentration (**Figure 2**). Statistically significant differences were observed between the highest concentration (CC_50_) of each extract compared with the LSA (*A. platensis*: *p*-value = 0.017; *D. tertiolecta*: *p*-value = 0.005).

In turn, the reference drugs also obtained a measurement of nitrite that was a little higher than the cells without stimulation and stimulated with LSA, but no proportionality between dosage and concentration was detected. For Sb^V^, a statistically significant difference was found between the concentrations of ½ CC_50_ (*p*-value = 0.028) and ¼ CC_50_ (*p*-value = 0.023) with the LSA, pointing out a higher NO induction of these concentrations compared to the antigen. Moreover, for MTF, a statistically significant difference was also found between the CC_50_ and LSA (*p*-value = 0.041).

### Gene Expression

From the stimulated cells, messenger RNA was extracted and reverse-transcribed to cDNA for quantification, targeting the genes *Tbx21*, *GATA3*, *RORc*, and *FOXP3*, which are related to the induction of T-cell differentiation for the Th1, Th2, Th17, and Treg profiles, respectively.

**Figure 3:**
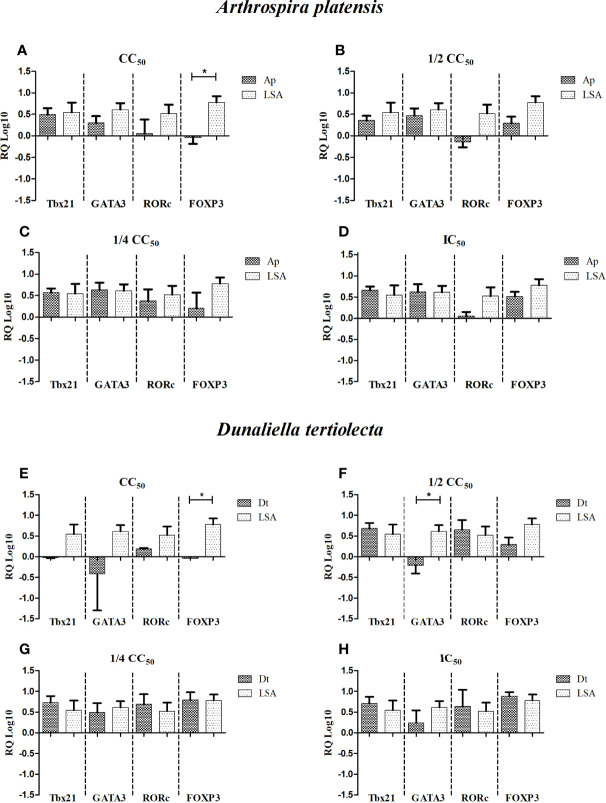
Relative quantification (RQ Log10) of gene expression of specific transcriptional factors after cell stimulation with bioactive extracts of photosynthetic microorganisms. **(A, E)** CC_50_ value; **(B, F)** half of the CC_50_ value; **(C, G)** one quarter of the CC_50_ value; **(D, H)** IC_50_ value. LSA, *Leishmania infantum* soluble antigen (25 µg/ml); Ap, *Arthrospira platensis*; Dt, *Dunaliella tertiolecta*. The calibrator used was phytohemagglutinin (PHA). *Statistical difference = *p*-value < 0.05.

When analyzing the results of gene expression stimulated with natural extracts (**Figure 3**), we observed that in all concentrations of *A. platensis* extract, a balance between the Th1/Th2 profile was present, with the average expression of the *Tbx21* gene being higher than that of *GATA3* at the highest stimulus concentration, with no statistical difference observed, similar to the results of gene expression stimulated with LSA. Notably, the *FOXP3* gene at the highest concentration showed a negative expression (**Figure 3A**), and the *RORc* gene varied from concentration to concentration, showing a greater expression at the ¼ CC_50_ concentration (**Figure 3C** **;** **Table 4**).

**Table 4:** Relative quantification (RQ Log10) of gene expression of specific transcriptional factors after cell stimulation with bioactive extracts of photosynthetic microorganisms.

Gene	CC_50_	½ CC_50_	¼ CC_50_	IC_50_
*Arthrospira platensis*				
*Tbx21*	0.49	0.36	0.57	0.67
*GATA3*	0.30	0.47	0.64	0.62
*RORc*	0.05	−0.14	0.38	0.05
*FOXP3*	−0.04	0.29	0.21	0.51
*Dunaliella tertiolecta*				
*Tbx21*	−0.02	0.69	0.73	0.71
*GATA3*	−0.41	−0.20	0.49	0.24
*RORc*	0.19	0.65	0.69	0.64
*FOXP3*	−0.05	0.29	0.79	0.88

RQ Log10 = 2^−ΔΔCt^. The calibrator used was phytohemagglutinin (PHA).

CC_50_, 50% cytotoxic concentration; IC_50_, 50% inhibitory concentration.

Regarding the extract of *D. tertiolecta*, we observed that at the lowest concentrations (**Figures 3G, H**), there was a balance between all the inducing genes, with the average expression of the *GATA3* gene being lower than the others. As shown by the results, the ½ CC_50_ concentration (**Figure 3F**) stimulated a balanced and positive expression between the Th1×Th17×Treg profiles, with a negative expression of *GATA3*, inducing the Th2 profile, and a statistically significant difference was observed when compared to LSA (*p*-value = 0.009). At the highest concentration, there was only a positive expression of the *RORc* gene (**Figure 3E**). Analyzing the correlation between genes, for *A. platensis*, a very strong positive correlation was observed between *Tbx21* and *RORc* at the ½ CC_50_ concentration (*r* = 0.95; *p*-value = 0.047). Regarding the correlation analysis of the extract of *D. tertiolecta*, at the concentrations tested, no significant correlations were detected. **Supplementary Table 3** shows the correlation data of gene expression induced by the extracts.

## Discussion

When searching for new therapeutic strategies, low toxicity to host cells is one of the important factors to be considered in identifying a potent therapeutic candidate (33). In this study, *A. platensis* extract demonstrated a higher CC_50_ value than the active principles of the drugs currently used for the treatment of VL and CVL (Glucantime^®^ and Milteforan™, respectively).

As alternative candidates for the development of therapeutics, photosynthetic marine organisms have been studied for their anti-*Leishmania* capacity; however, studies evaluating phytoplankton for their effect against *Leishmania* sp. are very scarce, being only reported by Pereira et al. (17), which evaluated the leishmanicidal activity of methanol extracts of four species of Chlorophyta, from three different genera (*Nannochloris*, *Picochlorum*, and *Desmochloris*). Therefore, further studies on the leishmanicidal effects of photosynthetic marine microorganisms are necessary, demonstrating the significance of this study. Thus, when comparing our results with those found by Pereira et al. (17), assessing the extracts of Chlorophyta species, we noted that the extracts of *D. tertiolecta* presented a lower IC_50_ value, that is, a greater leishmanicidal/leishmanistatic activity, since the concentration of *Nannochloris* sp. (250 µg/ml) that was used reduced the viability of *L. infantum* by 38%.

When observing the leishmanicidal/leishmanistatic activity of the natural extracts in this study, we noted that the IC_50_ values were lower when compared to the values of some organic plant extracts evaluated by Bouyahya et al. (34), with IC_50_ values ranging between 82.39 and 500 µg/ml, which were more toxic for *L. infantum*. Making a new comparison, this time with meroditerpenoid isolates from a brown macroalgae (*Cystoseira baccata*) evaluated by Sousa et al. (35), we observed in our study that the SI of the extract of *D. tertiolecta* was higher, since the SI obtained by the Sousa et al. was less than 10, using murine peritoneal macrophages, demonstrating that the extracts studied here may have promising potential.

Although the extracts from both *A. platensis* and *D. tertiolecta* studied here do not present an SI greater than 10 in assays with human cells, we do not exclude the immunomodulatory potential of these extracts. As observed in the study by Costa-Silva et al. (36), isolates from natural products, despite presenting an SI less than 10, had immunomodulatory activity against *L. infantum*, reducing the production of Th2 profile cytokines, in addition to stimulating chemokines associated with a decrease in the parasite load.

When evaluating the immune response induced by bioactive extracts of photosynthetic microorganisms of the species *A. platensis* and *D. tertiolecta*, a positive immune stimulus of the cells was observed. Different concentrations were studied, but all maintained the same general pattern of the immune response, a balance between pro-inflammatory and anti-inflammatory cytokines, with some concentrations having a slightly greater emphasis on the Th1 profile cytokines.

*Arthrospira platensis* is a cyanobacterium with some biological activities that have been known for some years by the scientific community. The immunomodulatory potential of this microorganism has been indicated in the literature, stimulating the production of IFN-γ and TNF and reducing the production of IL-10, both in mice (37) and in human PBMCs (38). In addition to the extract, phycocyanin (photosynthetic pigment), a protein isolated from *A. platensis*, studied by Sheikh et al. (39) when they performed PBMC treatment, also demonstrated an increase in IFN-γ. These findings corroborate our results, since we observed a greater production of IFN-γ and TNF compared to cells without stimulus, with special emphasis on IFN-γ that showed a greater synthesis compared to LSA, with a statistical difference in the lowest concentration.

In addition to stimulating the production of Th1 cytokines, the *A. platensis* extract also induced the synthesis of the leishmanicidal factor, strengthening the hypothesis that this extract is a good candidate for treating VL. The expression of specific transcriptional factor genes showed a balance, but with no statistically significant differences, strengthening the hypothesis that this extract stimulates the modulation of the immune response by expressing the *Tbx21* gene, responsible for activating the differentiation of Th1 lymphocytes.

Although *D. tertiolecta* is a halophilic microalgae widely used in food and feed supplementation, there are few studies reporting the immunological activity of this species. Based on these few studies about immune response, the immunological activity of this species was observed *in vitro* in PBMCs from sheep, making it possible to identify the ability of phytosterols from this species to stimulate an immune response. Among these phytosterols, 7-dehydroporiferasterol and ergosterol induced the production of the cytokine IL-10 and the reduction of IL-6 and TNF, demonstrating an anti-inflammatory role (40). Likewise, in the study by Caroprese et al. (41), an increase in IL-10 synthesis was observed, with a reduction in pro-inflammatory cytokines. In addition, a dose-dependent IL-10 was identified, which was also found in our study; however, in our results, we observed that with increasing concentration, IL-10 decreased.

In our study, the extract of *D. tertiolecta* presented a high stimulation of TNF, surpassing LSA, and a low synthesis of IL-10, lower than LSA, mainly at the highest concentrations tested. The diverging results presented in this study from those found in the literature may be due to the difference in the molecules: the extracts investigated in our study were protein-based, whereas those in the studies by Caroprese et al. (40) and Ciliberti et al. (41) were from phytosterol isolates.

The cytokines produced by the Th1 profile were directly related to the production of the leishmanicidal factor, which was found in our study, stimulating the synthesis of IFN-γ and TNF, in addition to the production of NO. These results also corroborated the data found in the analysis of gene expression of transcriptional factors, which showed the *Tbx21* gene to be a little more expressed than *GATA3* or *FOXP3*, depending on the concentration used.

The strong correlations (positive or negative) found by the stimulation with the extracts, both for cytokines and transcription factor genes, are interesting. Although not significant, a strong negative correlation found between TNF and IL-10 for the extract of *D. tertiolecta* stimulus was observed in the graphs and corroborated with the hypothesis that IL-10 production is dose-dependent. For *A. platensis*, we also observed very strong negative correlations, although not significant, between TNF and Th2 profile cytokines, suggesting an immunomodulatory potential. The very strong positive correlation between the TNF and IL-4 cytokines is also noteworthy, since the increase of a Th1 profile cytokine together with anti-inflammatory cytokines can combat the parasite, as the balance between both profiles (but predominantly Th1) is important to achieve disease control (42).

Regarding the correlation between genes stimulated by the *A. platensis* extract, the significant positive correlation found between *Tbx21* and *RORc*, transcriptional factors responsible for inducing Th1 and Th17 profiles, respectively, demonstrated the potential to stimulate pro-inflammatory cytokines, a significant aspect of the immune response against the VL parasite.

Finally, when comparing the results of the extracts with those of the reference drugs, we observed that MTF had a selectivity of more than 100 times for the parasite than for the cells of human and canine hosts, showing greater selectivity among all stimuli. On the other hand, Sb^V^ had the lowest SI value, demonstrating that all therapeutic candidates were more selective than Sb^V^. Furthermore, an immunological susceptibility profile was observed, unlike the extracts, demonstrating the induction of a protective profile.

In conclusion, these data suggest and demonstrate that the extracts of PMs are promising therapeutic candidates to treat visceral leishmaniasis, with a protective response elicited in the human cells. These extracts will be further evaluated in relation to their ability i) to stimulate parasitological cure in *in-vivo* assays (preclinical trials), ii) to induce an immune response, and iii) to reduce the parasite load, and their action on amastigotes will also be analyzed.

## Data Availability Statement

The original contributions presented in the study are included in the article/**Supplementary Material**. Further inquiries can be directed to the corresponding author.

## Ethics Statement

The studies involving human participants were reviewed and approved by Aggeu Magalhães Institute - Fiocruz Pernambuco. The patients/participants provided their written informed consent to participate in this study.

## Author Contributions

VV-A planned and performed the experiments, analyzed the data, and wrote the manuscript. JS, IR, and MM helped in carrying out the experiments. JS and AA were responsible for the cultivation of the photosynthetic microorganisms and obtaining the extracts. PA collaborated with referral drugs. RB, DM, and SS wrote sections of the manuscript. RS, VL, and MP contributed substantially to the data analysis and assisted in the writing of the manuscript. All authors contributed to the review of the manuscript and read and approved the submitted version.

## Funding

This study was partially financed by the Coordination for the Improvement of Higher Education Personnel – Brazil (CAPES) (Finance Code 001), the Research Excellence Program (CNPq/PROEP/FIOCRUZ) (400711/2019-2), the National Council for Scientific and Technological Development (CNPq) (PQ 301223/2018.1), and the Foundation for Science and Technology Pernambuco (FACEPE) (Finance Code PBPG – 13666-2.11/18).

## Conflict of Interest

The authors declare that the research was conducted in the absence of any commercial or financial relationships that could be construed as a potential conflict of interest.

## Publisher’s Note

All claims expressed in this article are solely those of the authors and do not necessarily represent those of their affiliated organizations, or those of the publisher, the editors and the reviewers. Any product that may be evaluated in this article, or claim that may be made by its manufacturer, is not guaranteed or endorsed by the publisher.
