# Supplementary material for: Therapeutic Potential of Photosynthetic Microorganisms for Visceral Leishmaniasis: An Immunological Analysis
Source: Front Immunol. 2022 Jun 30;13:891495. doi: 10.3389/fimmu.2022.891495 (PMC9280147; doi:10.3389/fimmu.2022.891495)
Supplement: Supplementary file 1 [file DataSheet_1.docx]

Supplementary Material

**Supplementary Table 1.** Correlation among the cytokines produced after the stimulus with the different concentrations of the extract of *Arthrospira platensis* and *Dunaliella tertiolecta*.

|  | **A x B** | **A x C** | **A x D** | **A x E** | **B x C** | **B x D** | **B x E** | **C x D** | **C x E** | **D x E** |  |
| --- | --- | --- | --- | --- | --- | --- | --- | --- | --- | --- | --- |
| *Arthrospira platensis* | | | | | | | | | | | |
| **CC_50_** | | | | | | | | | | | |
| r | 0.65 | 0.57 | -0.50 | -0.18 | 0.40 | -0.92 | 0.10 | -0.41 | -0.83 | 0.05 |  |
| p-value | 0.24 | 0.32 | 0.39 | 0.77 | 0.50 | 0.02* | 0.87 | 0.50 | 0.08 | 0.93 |  |
| **1/2 CC_50_** | | | | | | | | | | | |
| r | 0.64 | 0.38 | 0.46 | -0.14 | 0.47 | 0.97 | 0.52 | 0.50 | 0.11 | 0.71 |  |
| p-value | 0.25 | 0.53 | 0.43 | 0.83 | 0.42 | 0.01* | 0.37 | 0.39 | 0.86 | 0.18 |  |
| **1/4 CC_50_** | | | | | | | | | | | |
| r | 0.23 | 0.12 | 0.46 | -0.59 | 0.38 | 0.30 | 0.13 | 0.86 | -0.41 | -0.37 |  |
| p-value | 0.71 | 0.85 | 0.44 | 0.30 | 0.53 | 0.63 | 0.84 | 0.06 | 0.49 | 0.54 |  |
| **IC_50_** | | | | | | | | | | | |
| rs | 0.10 | 0.20 | 0.70 | -0.40 | -0.40 | 0.00 | -0.20 | 0.70 | -0.60 | -0.90 |  |
| p-value | 0.95 | 0.78 | 0.23 | 0.52 | 0.52 | - | 0.78 | 0.23 | 0.35 | 0.08 |  |
| *Dunaliella tertiolecta* | | | | | | | | | | | |
| **CC_50_** | | | | | | | | | | | |
| *rs* | 0.70 | 0.30 | 0.50 | 0.60 | 0.30 | 0.30 | 0.90 | 0.70 | 0.10 | 0.40 |  |
| *p-value* | 0.23 | 0.68 | 0.45 | 0.35 | 0.68 | 0.68 | 0.08 | 0.23 | 0.95 | 0.52 |  |
| **1/2 CC_50_** | | | | | | | | | | | |
| *rs* | 0.00 | -0.30 | -0.30 | 0.30 | 0.20 | 0.20 | -0.70 | -0.20 | -0.70 | 0.30 |  |
| *p-value* | - | 0.68 | 0.68 | 0.68 | 0.78 | 0.78 | 0.23 | 0.78 | 0.23 | 0.68 |  |
| **1/4 CC_50_** | | | | | | | | | | | |
| *rs* | -0.60 | -0.20 | 0.30 | 0.40 | -0.60 | -0.50 | -0.50 | 0.30 | -0.10 | 0.00 |  |
| *p-value* | 0.35 | 0.78 | 0.68 | 0.52 | 0.35 | 0.45 | 0.45 | 0.68 | 0.95 | - |  |
| **IC_50_** | | | | | | | | | | | |
| *rs* | 0.40 | -0.90 | -0.60 | 0.30 | -0.70 | -0.30 | -0.10 | 0.70 | -0.10 | 0.50 |  |
| *p-value* | 0.52 | 0.08 | 0.35 | 0.68 | 0.23 | 0.68 | 0.95 | 0.23 | 0.95 | 0.45 |  |

A (IFN-γ); B (TNF); C (IL-10); D (IL-4); E (IL-2); r (Pearson's correlation coefficient); rs (Spearman's correlation coefficient); CC_50_ (50% Cytotoxic Concentration); IC_50_ (50% Inhibitory Concentration); * (Statistical difference = p-value < 0.05); - (not determined).

**Supplementary Table 2.** Correlation among the cytokines produced after the stimulus with the different concentrations of the n-Methylglucamine Antimoniate and Miltefosine.

|  | | **A x B** | | **A x C** | | **A x D** | | **A x E** | | **B x C** | | **B x D** | | **B x E** | | **C x D** | | **C x E** | | **D x E** |  |
| --- | --- | --- | --- | --- | --- | --- | --- | --- | --- | --- | --- | --- | --- | --- | --- | --- | --- | --- | --- | --- | --- |
| **n-Methylglucamine Antimoniate** | | | | | | | | | | | | | | | | | | | | |  |
| **CC_50_** | | | | | | | | | | | | | | | | | | | | |  |
| *rs* | | 0.00 | | 0.40 | | - | | 0.80 | | -0.20 | | 0.00 | | -0.40 | | -0.40 | | 0.00 | | -0.80 |  |
| *p-value* | | - | | 0.75 | | - | | 0.33 | | 0.92 | | - | | 0.75 | | 0.75 | | - | | 0.33 |  |
| **1/2 CC_50_** | | | | | | | | | | | | | | | | | | | | |  |
| *r* | | -0.50 | | 0.69 | | -0.54 | | 0.74 | | 0.22 | | 0.88 | | -0.11 | | 0.23 | | 0.90 | | 0.09 |  |
| *p-value* | | 0.50 | | 0.31 | | 0.46 | | 0.26 | | 0.78 | | 0.12 | | 0.89 | | 0.77 | | 0.10 | | 0.91 |  |
| **1/4 CC_50_** | | | | | | | | | | | | | | | | | | | | |  |
| *r* | | -0.81 | | 0.62 | | -0.64 | | 0.01 | | -0.89 | | 0.91 | | 0.52 | | -1.00 | | -0.41 | | 0.43 |  |
| *p-value* | | 0.19 | | 0.38 | | 0.36 | | 0.99 | | 0.11 | | 0.09 | | 0.48 | | 0.001 | | 0.59 | | 0.57 |  |
| **IC_50_** | | | | | | | | | | | | | | | | | | | | |  |
| *rs* | | -0.80 | | 0.80 | | - | | 0.20 | | - | | 0.80 | | -0.40 | | -0.80 | | 0.40 | | -0.20 |  |
| *p-value* | | 0.33 | | 0.33 | | - | | 0.92 | | - | | 0.33 | | 0.75 | | 0.33 | | 0.75 | | 0.92 |  |
| **Miltefosine** | | | | | | | | | | | | | | | | | | | | | |
| **CC_50_** | | | | | | | | | | | | | | | | | | | | | |
| *r* | 0.05 | | 0.72 | | -0.22 | | 0.46 | | 0.71 | | 0.22 | | -0.18 | | -0.14 | | 0.32 | | -0.97 | | |
| *p-value* | 0.95 | | 0.28 | | 0.78 | | 0.54 | | 0.29 | | 0.78 | | 0.82 | | 0.86 | | 0.68 | | 0.03* | | |
| **1/2 CC_50_** | | | | | | | | | | | | | | | | | | | | | |
| *rs* | 0.00 | | 0.80 | | 0.00 | | 1.00 | | 0.60 | | 1.00 | | 0.00 | | 0.60 | | 0.80 | | 0.00 | | |
| *p-value* | - | | 0.33 | | - | | 0.08 | | 0.42 | | 0.08 | | - | | 0.42 | | 0.33 | | - | | |
| **1/4 CC_50_** | | | | | | | | | | | | | | | | | | | | | |
| *rs* | 0.40 | | 0.70 | | -0.43 | | 0.53 | | 0.93 | | 0.50 | | -0.08 | | 0.23 | | 0.13 | | -0.88 | | |
| *p-value* | 0.60 | | 0.30 | | 0.57 | | 0.47 | | 0.07 | | 0.50 | | 0.92 | | 0.77 | | 0.87 | | 0.12 | | |
| **IC_50_** | | | | | | | | | | | | | | | | | | | | | |
| *r* | -0.59 | | 0.64 | | 0.15 | | 0.61 | | -0.99 | | 0.54 | | -0.78 | | -0.44 | | 0.74 | | -0.68 | | |
| *p-value* | 0.41 | | 0.36 | | 0.85 | | 0.39 | | 0.01* | | 0.46 | | 0.22 | | 0.56 | | 0.26 | | 0.32 | | |

A (IFN-γ); B (TNF); C (IL-10); D (IL-4); E (IL-2); r (Pearson's correlation coefficient); rs (Spearman's correlation coefficient); CC_50_ (50% Cytotoxic Concentration); IC_50_ (50% Inhibitory Concentration); * (Statistical difference = p-value < 0.05); - (not determined).

**Supplementary Table 3.** Correlation among the gene expression induced after the stimulus with the different concentrations of the extract of *Arthrospira platensis* and *Dunaliella tertiolecta*.

|  | **A x B** | **A x C** | **A x D** | **B x C** | **B x D** | **C x D** |
| --- | --- | --- | --- | --- | --- | --- |
| *Arthrospira platensis* | | | | | | |
| **CC_50_** | | | | | | |
| r | -0.18 | 0.50 | -0.43 | 0.76 | -0.97 | -0.90 |
| p-value | 0.89 | 0.67 | 0.57 | 0.45 | 0.16 | 0.29 |
| **1/2 CC_50_** | | | | | | |
| r | -0.22 | 0.95 | 0.87 | -0.28 | 0.24 | 0.84 |
| p-value | 0.78 | 0.047* | 0.054 | 0.72 | 0.76 | 0.16 |
| **1/4 CC_50_** | | | | | | |
| r | -0.48 | -0.85 | 0.72 | 0.97 | 0.28 | -0.21 |
| p-value | 0.52 | 0.35 | 0.17 | 0.17 | 0.72 | 0.87 |
| **IC_50_** | | | | | | |
| r | -0.41 | -0.23 | 0.59 | 0.88 | 0.14 | 0.55 |
| p-value | 0.59 | 0.77 | 0.29 | 0.12 | 0.86 | 0.45 |
| *Dunaliella tertiolecta* | | | | | | |
| **CC_50_** | | | | | | |
| r | - | - | - | - | - | - |
| p-value | - | - | - | - | - | - |
| **1/2 CC_50_** | | | | | | |
| r | 0.35 | - | 0.94 | - | 0.02 | 0.45 |
| p-value | 0.77 | - | 0.21 | - | 0.99 |  |
| **1/4 CC_50_** | | | | | | |
| r | -0.87 | - | -0.96 | - | 0.97 | - |
| p-value | 0.32 | - | 0.17 | - | 0.16 | - |
| **IC_50_** | | | | | | |
| r | -0.68 | 0.95 | -0.76 | -0.81 | 0.91 | -0.91 |
| p-value | 0.32 | 0.20 | 0.24 | 0.40 | 0.09 | 0.27 |

A (Tbx21); B (GATA3); C (RORc); D (FOXP3); r (Pearson's correlation coefficient); CC_50_ (50% Cytotoxic Concentration); IC_50_ (50% Inhibitory Concentration); * (Statistical difference = p-value < 0.05); - (not determined).
